# Attending to Race (or Gender) Does Not Increase Race (or Gender) Aftereffects

**DOI:** 10.3389/fpsyg.2016.00909

**Published:** 2016-06-17

**Authors:** Nicolas Davidenko, Chan Q. Vu, Nathan H. Heller, John M. Collins

**Affiliations:** Department of Psychology, University of California, Santa Cruz, Santa CruzCA, USA

**Keywords:** face adaptation, attention, race, gender, aftereffects

## Abstract

Recent research has shown that attention can influence the strength of face aftereffects. For example, attending to changes in facial features increases the strength of identity and figural aftereffects relative to passive viewing (Rhodes et al., 2011). Here, we ask whether attending to a specific social dimension of a face (such as race or gender) influences the strength of face aftereffects along that dimension. Across three experiments, participants completed many single-shot face adaptation trials. In each trial, participants observed a computer-generated adapting face for 5 s while instructed to focus on either the race or gender of that adapting face. Adapting faces were either Asian and female or Caucasian and male. In Experiment 1, all trials included an intermediate question (IQ) following each adaptation period, soliciting a rating of the adapting face on the attended dimension (e.g., race). In Experiment 2, only half of the trials included this IQ, and in Experiment 3 only a quarter of the trials did. In all three experiments, participants were subsequently presented with a race- and gender-neutral face and asked to rate it on either the attended dimension (e.g., race, *attention-congruent trials*) or the unattended dimension (e.g., gender, *attention-incongruent trials*) using a seven-point scale. Overall, participants showed significant aftereffects in all conditions, manifesting as (i) higher Asian ratings of the neutral faces following Caucasian vs. Asian adapting faces and (ii) higher female ratings of neutral faces following male vs. female adapting faces. Intriguingly, although reaction times were shorter during attention-congruent vs. attention-incongruent trials, aftereffects were not stronger along attention-congruent than attention-incongruent dimensions. Our results suggest that attending to a facial dimension such as race or gender does not result in increased adaptation to that dimension.

## Introduction

For almost 20 years, understanding face adaptation has been a central focus of face perception research. As with motion and color adaptation, face adaptation has been shown to shift perceptual ratings for certain facial characteristics or dimensions, and it is thought to function by retuning populations of neurons that code for those features (Yang et al., 2011). Adapting to a face for several seconds or minutes results in systematic shifts in the perception of subsequently viewed faces, known as figural aftereffects (AEs; Webster and Maclin, 1999; Webster and MacLeod, 2011). These high-level AEs depend on the characteristics of the adapting face; for instance, adapting to a face with male characteristics leads participants to rate a neutral face as appearing more female. A wide variety of facial characteristics can be adapted to, including identity, gender, race, age, expression, eye gaze, and figural distortions (Hsu and Young, 2004; Webster et al., 2004; Rhodes and Jeffery, 2006; Seyama and Nagayama, 2006; Davidenko et al., 2008; Little et al., 2008). The current study investigates whether attention can influence which facial characteristics become most adapted to. We begin more generally by reviewing how visual attention and face perception interact.

Palermo and Rhodes (2007) composed a comprehensive review of studies examining interactions between face perception and attention, classifying them into two broad categories: studies showing that certain automatic processes in face perception, such as detection, occur in the absence of attention; and studies examining the role of selective attention in modulating more detailed face processing tasks, such as preferences for expressive faces. In the former category, Driver et al. (1999) demonstrated that simply observing an image of a face that is gazing either left or right influences the reaction time to targets appearing next to the face, with faster reactions to targets that were congruent with the gaze. Even when participants were told that the targets were four times more likely to appear on the side opposite the gaze direction, participants were still driven to respond congruently with the gaze. These findings strongly support the claim that eye gaze is processed automatically and leads to attentional shifts, even when participants actively attempt to ignore it. However, a subsequent study by Ristic and Kingstone (2005) showed that these gaze cueing effects only take place when a person consciously recognizes the stimulus as a face. By using an ambiguous stimulus that could be seen as either the front of a car or as gazing eyes, the authors showed that it was only when participants were explicitly told that the cue was a pair of eyes that they were reflexively driven by gaze direction. These studies show that certain facial characteristics can be processed automatically, but rely on face detection having already taken place. Thus, aspects of face perception can be both immune to and beholden to attention.

As Palermo and Rhodes (2002) point out in their review, the relationship between attention and face perception is complex and depends on (i) the aspect of face perception being considered, (ii) the facial features being processed, and (iii) the type of attentional task being employed. For instance, a study conducted by the same authors (Palermo and Rhodes, 2002) tested how holistic face processing might be modulated by attention. The holistic processing account posits that faces are perceived in their entirety, rather than as an aggregate of their parts (Tanaka and Farah, 1993). Palermo and Rhodes (2002) used the part-whole task (Tanaka and Farah, 1993), where participants had to identify parts of faces that they have seen before, either in isolation or incorporated into a whole-face image. In addition, flanker faces appeared on either side of the target stimuli. Half of participants were told they would later have to match the identity of the flanker faces, and the other half were not. The authors found that holistic face processing (manifesting as better performance in whole-face trials) was intact for participants who could ignore the flankers, but was impaired for participants who attended to the flankers, showing that attending to face identity co-opted attentional resources and suppressed holistic processing. However, a recent study by Norman and Tokarev (2014) also investigated the relationship between attention and holistic processing using different methods and found different results. In their study, the authors measured whether the composite face effect (Young et al., 1987) was modulated by the presentation of an exogenous cue. On half of the trials, participants’ attention was drawn away from the test face and on the other half attention was made to align with the test face. The authors found no differences in holistic processing under these conditions, and thus concluded that spatial attention does not impact the attentional resources needed for holistic face processing. Therefore, even the same aspect of face perception (holistic processing) can interact differently with attention depending on the attentional task itself.

Similar complexities emerge regarding how attention modulates perception of different facial characteristics, such as gender, race, expression, and attractiveness. Reddy et al. (2004) carried out a study where they pitted a demanding letter discrimination task against a gender discrimination task and found that their participants could accurately tell the gender of a face even while attending to the letters. However, Murray et al. (2011) challenged the view that this sort of feature discrimination is entirely independent of attention. In their study, they varied the perceptual load of a word search task, requiring participants to devote more or less attention to it, and then asked them to discriminate the race or gender of a task-irrelevant face that was presented peripherally. They showed that under low attentional load, the race or gender of the peripheral face was indeed processed automatically, but as the perceptual load increased, the race or gender of a face was effectively ignored.

Relatively less is known regarding the effects of attention on face adaptation. One recent study carried out by Murray et al. (2012) found that figural face AEs occurred even when participants attended to a letter/color discrimination task and adapting faces were presented peripherally. Their study demonstrated that like other aspects of face perception, figural face AEs can take place even when adapting faces are not being attended to. Nevertheless, an earlier study conducted by Moradi et al. (2005) established that identity AEs are only observed when observers are explicitly aware of the adapting face. Using a binocular rivalry technique, the authors showed that when an adapting face is successfully suppressed for the whole trial, adaptation does not take place. Thus similarly to the gaze cueing effect, face adaptation is in some cases automatic and occurs without explicit attention, and yet it is dependent on some degree of conscious perception.

Rhodes et al. (2011) were the first to demonstrate a positive effect that attention can have on face AEs. The authors conducted two experiments, each with a different adapting category and a different attentional task. In the first experiment, they examined identity AEs while participants detected whether or not the lips or eyes of the adapting face became lighter in shade; in the second experiment, they examined figural AEs while participants performed a one-back task with the adapting faces. Each experiment also included a passive-viewing condition. In both experiments, the authors found that AEs were greater during the attention condition than during the passive-viewing condition, demonstrating that attending to specific features or to the identity of a face increases the degree of adaptation relative to passive viewing.

In the present study, we ask the following question: does attending to one facial characteristic of a face influence how another facial characteristic is adapted to? Whereas previous studies considered how the presence or absence of attention modulates adaptation more generally, our study addresses how the specific dimension of attention impacts that specific dimension of adaptation. We conducted three experiments that compared how attending to race or gender impacts adaptation to race or gender. In each experiment, we asked participants to focus on either the race or gender of an adapting face, and then measured subsequent AEs in the race and gender dimensions.

## Materials and Methods

### Participants

Participants were 199 University of California, Santa Cruz undergraduates (ages 18–23; 141 female) who gave informed consent and participated for course credit. The University of California, Santa Cruz IRB approved the experimental paradigm. There were 54 participants in Experiment 1, 91 in Experiment 2, and 54 in Experiment 3.

### Stimuli

**FIGURE 1:**
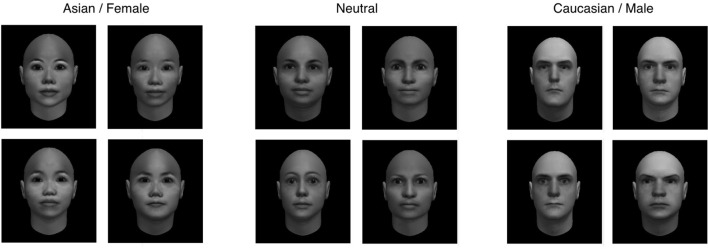
**Example stimuli from Experiments 1 and 2.** Left panel: Asian female adapting faces; center panel: race- and gender-neutral test faces; right panel: Caucasian male adapting faces.

We used FaceGen Modeller 3.1^1^ to generate faces varying across dimensions of race and gender. To construct each face, we started with a random initial face and adjusted the race and gender scales to generate 12 Asian female and 12 Caucasian male faces. For Experiments 1 and 2, we similarly generated *neutral* faces by starting with 12 random initial faces, adjusting the gender scale to a perceptually neutral level, and matching the contribution of Caucasian and Asian races while minimizing the contribution of other races. Example stimuli are shown in **Figure 1**. We found that faces generated on the midpoint of FaceGen’s gender scale appeared more in male than female. Therefore to generate a set of faces that were as neutral as possible on both the race and gender dimensions, we conducted a short calibration study soliciting ratings from 12 research assistants in our lab who were naive to the hypothesis of our study. In the calibration study, participants were presented with a superset of 48 potential neutral faces with instructions to rate each one on a seven-point race or gender scale. Based on these results, we selected the set of 12 neutral faces that elicited the most balanced ratings of race and gender.

For Experiment 3, we constructed neutral faces in a different way, by creating morphs between pairs of adapting faces. Because 50% morphs between arbitrary pairs of adapting faces did not typically result in neutral faces on these dimensions, we adjusted the morph level for each face until it appeared as neutral as possible (see **Figure 5**). To validate the neutral faces, we conducted a short calibration study on 19 new participants recruited from University of California, Santa Cruz. In the calibration study, participants were presented with a randomly chosen adapting face (either Asian female or Caucasian male) for 5 s, with instructions to focus on the face. After each adapting face, a neutral face was displayed for 250 ms and participants were asked to rate it on either race or gender, using a seven-point scale. Based on the results, the initial set of 12 neutral faces was parsed down to 8 faces that elicited the most balanced ratings of race and gender across all adapting faces.

In all three experiments, the face images were converted to gray scale to eliminate the potential use of color information for judgments of race and gender, and presented on a black background.

### Procedure

#### General Procedure

In Experiments 1, 2, and 3, participants sat approximately 18″ away from a 21-inch LCD screen, where face images subtended approximately 7**°**×7**°** of visual angle. The experiment presentation and data collection scripts were written in Matlab. Before each experiment began, a research assistant read the instructions out loud, asking participants to pay close attention to faces that would be displayed and to follow the focusing instructions. Participants were not instructed to provide responses quickly. In each trial, participants observed a randomly chosen adapting face for 5 s with overlaid instructions to focus on either the race or the gender of the face (these instructions appeared 700 ms prior to the presentation of the face Experiments 2 and 3 and remained present throughout the subsequent 5 s). After a 700 ms black screen, participants were then presented with an IQ asking them to rate that adapting face on a seven-point scale. For *attend-race* trials, the rating scale ranged from 1 (extremely Asian) to 7 (extremely Caucasian). For *attend-gender* trials, the rating scale ranged from 1 (extremely female) to 7 (extremely male). Following the participant’s response, a black screen appeared for 300 ms, followed by a presentation of a randomly chosen neutral face for 300 ms, followed by another black screen prompting participants to rate that neutral face on either race or gender, using the corresponding seven-point scale. Half of the trials were *attention-congruent* (e.g., participants attended to the *race* of the adapting face and then rated the *race* of the neutral face) and half were *attention-incongruent* (e.g., participants attended to the *gender* of the adapting face but rated the *race* of the neutral face).

##### Experiment 1 Procedure

In Experiment 1, 54 participants completed 96 trials where they adapted to a face and rated a subsequent face by typing a number from 1 to 7 on a keyboard. On race trials, 1 corresponded to “extremely Asian” and 7 to “extremely Caucasian”; on gender trials, 1 corresponded to “extremely female” and 7 to “extremely male”. The IQ regarding the race or gender of the adapting face was presented in every trial.

##### Experiment 2 Procedure

In Experiment 2, 91 participants completed 160 trials similar to those in Experiment 1, except that they entered their ratings by clicking on a seven-point scale on the screen. To more clearly dissociate the race and gender scales, we presented the race scale horizontally (with “extremely Asian” on the far left and “extremely Caucasian” on the far right) and the gender scale vertically (with “extremely female” on the top of the scale and “extremely male” on the bottom). In this experiment, the IQ was presented on only 50% of trials (in the other 50%, the neutral test face was presented immediately following the black screen after the adapting face).

##### Experiment 3 Procedure

In Experiment 3, 54 participants completed 160 trials identical to those in Experiment 2, except that the neutral faces were constructed differently (see Stimuli and **Figure 4**) and the IQ was presented on only 25% of trials.

## Results

In the three experiments, we present results averaged across participants with error bars showing standard error of the mean. For reaction time data, we computed each participant’s median reaction time in each type of trial, and we present the mean across participants along with the standard error of the mean. To quantify AEs for race and gender, we subtracted ratings of neutral faces following the two types of adapting faces. For example, to measure gender AEs, we subtracted gender ratings of neutral faces following Asian female adapting faces from those following Caucasian male adapting faces. The aftereffect-consistent difference measures (AE) for race and gender are therefore defined as follows:

$${AE}_{Race}=R_{Neutral}\left(adapt\,\;Asian\right)-R_{Neutral}\left(adapt\,\;Caucasian\right)$$

$${AE}_{Gen\,der}=G_{Neutral}\left(adapt\,\;female\right)-G_{Neutral}\left(adapt\,\;male\right)$$

where *R*_Neutral_ is the average race ratings of neutral faces, and *G*_Neutral_ is the average gender ratings of neutral faces.

### Experiment 1 Results

**FIGURE 2:**
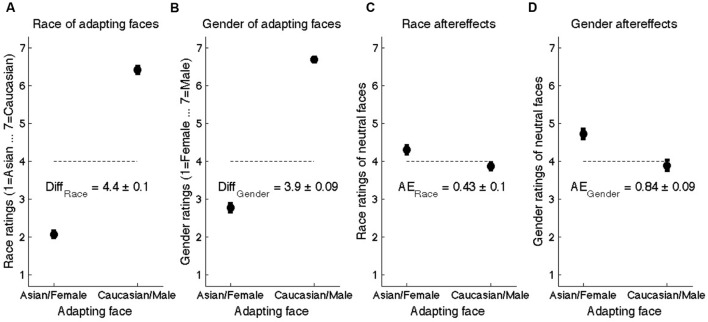
**Results of Experiment 1.** **(A)** Mean race ratings of adapting faces on a seven-point scale where 1 = extremely Asian and 7 = extremely Caucasian. **(B)** Mean gender ratings of adapting faces on a seven-point scale where 1 = extremely female and 7 = extremely male. **(C)** Mean race ratings of neutral faces as a function of the adapting face. **(D)** Mean gender ratings of neutral face as a function of the adapting face. “AE” refers to the aftereffect-consistent difference between ratings as a function of the adapting face. Error bars denote standard error of mean across participants.

Based on responses to the IQ, we confirmed that the adapting faces were unambiguous on the race and gender dimensions. On average, Asian female faces were rated as 2.07 ± 0.09 on the seven-point race scale and as 2.77 ± 0.11 on the seven-point gender scale; Caucasian male faces were rated as 6.42 on the race scale and 6.69 on the gender scale (see **Figures 2A,B**). We did observe a significant *male bias* in these ratings, wherein male adapting faces were rated as significantly more male than female adapting faces were rated as female (**Figure 2B**). We noted that similar male biases have been reported in the rating human faces (e.g., Davidenko, 2007, in the perception of face silhouettes; Gaetano et al., 2016, in the perception of faces and hands under a variety of formats), and Bayesian models have been proposed to explain these biases (Clifford et al., 2015). Nevertheless, the variable of interest here was the *difference* in ratings between the different adapting categories, regardless of any general biases. Overall, the difference in race ratings between adapting faces (4.35 ± 0.17) was slightly larger than the difference in gender ratings between adapting faces (3.92 ± 0.14; *t*), although this difference was small. Based on previous work that shows a monotonic relationship between the extremeness of adapting faces and the strength of aftereffects (e.g., Jeffery et al., 2011), we predicted that aftereffects for race and gender would be comparable (or if anything, race aftereffects would be slightly stronger than gender aftereffects).

To quantify race and gender aftereffects (AEs), we compared ratings of neutral faces following the two types of adaptors. Mean race ratings of neutral faces were 4.30 ± 0.11 following Asian female adaptors, compared to 3.88 ± 0.10 following Caucasian male adaptors (AE_Race_: 0.43 ± 0.09). Mean gender ratings of neutral faces were 4.73 ± 0.12 following Asian female adaptors, compared to 3.89 ± 0.14 following Caucasian male adaptors (AE_Gender_: 0.84 ± 0.12; see **Figures 2C,D**). Overall, AEs were significantly stronger for gender than for race AEs (*t*_53_ = 4.28, *p* < 0.0001), a difference we did not predict and that we address in Experiment 3.

**FIGURE 3:**
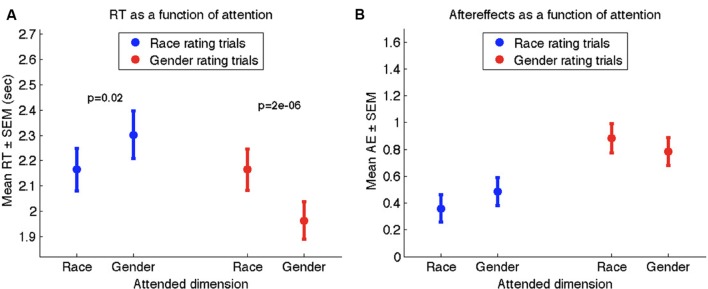
**Results of Experiment 1 as a function of attention.** **(A)** Mean reaction times (in s) to rate the neutral face on race (blue) or gender (red) as a function of the attended dimension during adaptation. **(B)** Mean aftereffect-consistent difference (AE) in ratings of race (blue) and gender (red) as a function of attended dimension during adaptation. Error bars denote standard error of the mean across 54 participants.

The question of interest was whether the attention manipulation (i.e. “focus on the race” or “focus on the gender”) influenced adaptation. To validate the effectiveness of the attention manipulation itself, we compared reaction times to rate the neutral face during *attention-congruent* vs. *attention-incongruent* trials. For race rating trials, mean RTs to rate the neutral face were significantly shorter when participants had been asked to focus on the race (2.16 s ± 0.08) vs. the gender (2.30 s ± 0.09) of the adapting face (mean difference: 0.14 s ± 0.06; *t*_53_ = 2.41, *p* < 0.05). Similarly, reaction times during gender rating trials were significantly shorter when attending to the gender (1.96 s ± 0.07) vs. the race (2.17 s ± 0.08) of the adapting face (mean difference: 0.20 s ± 0.04; *t*_53_ = 5.36, *p* < 0.00001; see **Figure 3A**). Collapsing across race and gender trials, participants were significantly faster to respond during attention-congruent trials (2.06 s ± 0.08) than during attention-incongruent trials (2.23 ± 0.08; *t* = 4.83, *p* < 0.0001).

Although the attention manipulation strongly influenced reaction times, it had no influence on the strength of AEs. Race AEs were no larger when participants had attended to the race (AE_Race_attend_race_: 0.36 ± 0.10) vs. the gender (AE_Race_attend_gender_: 0.49 ± 0.10) of the adapting face (mean difference: 0.13 +0.10; *t*_53_ = –1.33, *p* > 0.1). Likewise, gender AEs during attend-gender trials (AE_Gender_attend_gender_ 0.78 ± 0.14) were not larger than those during attend-race trials (AE_Gender_attend_race_ 0.88 ± 0.11; difference: 0.10 +0.11; *t*_53_ = –0.90, *p* > 0.1; see **Figure 3B**). In fact, there was a non-significant negative trend when collapsing across race and gender trials, wherein attention-incongruent trials elicited slightly stronger AEs (0.68 ± 0.09) than attention-congruent trials (0.57 ± 0.11; *t*_53_ = –1.58, *p* = 0.12).

To account for this unexpected trend, we considered the possibility that the IQ itself may have led to a response repetition bias, which would counteract the adaptation AEs during attention-congruent trials. For example, a participant responding to the IQ about the race of an adapting Caucasian face might accidentally repeat that response when asked about the race of the neutral face. In contrast, during attention-incongruent trials, the second question would be about the gender of the neutral face, eliminating the possibility of a response repetition bias. Because such a mechanism would weaken the apparent strength of AEs specifically during attention-congruent trials, it may conceal any attention-related enhancement in AEs. For this reason, in Experiments 2 and 3 we interspersed trials with and without the IQ to examine its possible role in the adaptation process and whether this ironic trend persists in the absence of an IQ. In addition, to further disambiguate responses along the two dimensions, participants in Experiments 2 and 3 entered their responses by clicking on one of two perpendicular scales on the screen (a horizontal scale for race and a vertical scale for gender), rather than by inputting numbers on the keyboard. This served to more clearly distinguish the two scales for participants and to provide a more sensitive response measure for each rating.

### Experiment 2 Results

**FIGURE 4:**
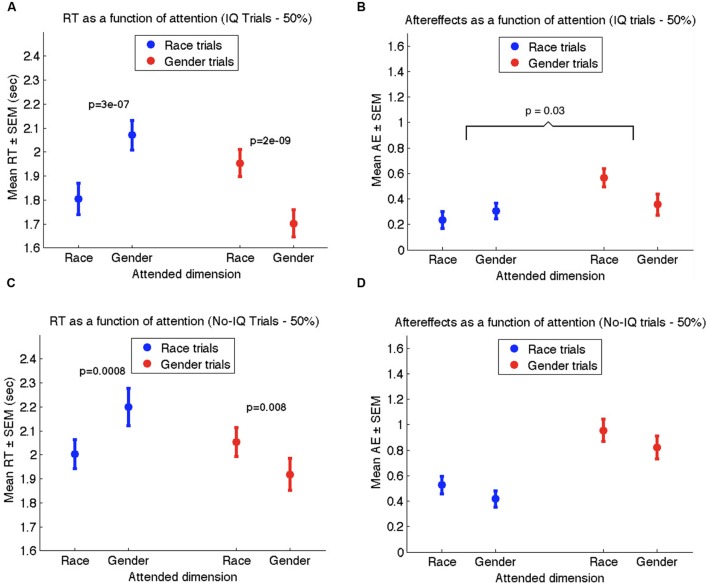
**Results of Experiment 2 for IQ trials **(A,B)** and no-IQ trials **(C,D)**.** **(A,C)** Mean reaction times to the neutral face during race rating trials (blue) and gender rating trials (red) as a function of the attended dimension. **(B,D)** Mean aftereffect-consistent difference (AE) in ratings of race (blue) and gender (red) as a function of attended dimension during adaptation. Error bars denote standard error of the mean across 91 participants.

We present results separately for IQ trials (those that included the IQ regarding the adapting face) and No-IQ trials (those with no IQ). In IQ trials, the attention instructions strongly influenced reaction times, just as in Experiment 1 (see **Figure 4A**). Ratings of neutral faces took significantly less time during attention-congruent trials (1.75 s ± 0.06) than during attention-incongruent trials (2.01 s ± 0.06; *t*_90_ = 8.06, *p* < 0.00001), serving to validate the attention manipulation. However, AEs were not stronger during attention-congruent trials (0.30 ± 0.06) than during attention-incongruent trials (0.44 ± 0.04). In fact, overall AEs were *weaker* in attention-congruent trials (*t*_90_ = –2.19, *p* = 0.03; see **Figure 4B**), corroborating the ironic trend we had observed in Experiment 1.

In No-IQ trials, the attention manipulation had a smaller but still significant effect on reaction times to neutral face, with a mean of 1.96 s ± 0.06 for attention-congruent trials and 2.13 s ± 0.06 for attention-incongruent trials (*t*_90_ = 4.54, *p* < 0.0001; **Figure 4C**), validating the effectiveness of the attention manipulation even in the absence of an IQ. Unlike reaction times, the magnitude of AEs was not influenced by the attention instructions, with overall AEs of 0.67 ± 0.06 for attention-congruent trials and 0.69 ± 0.06 for attention-incongruent trials (non-significant difference, *p* > 0.5; **Figure 4D**). Importantly, when the IQ was not present, there was no ironic effect of attention on AEs.

By considering both IQ and No-IQ trials in the same experiment, we were able to characterize the effect of the IQ on adaptation process. Including the IQ led to significantly weaker overall AEs (0.37 ± 0.04) compared to not including the IQ (0.68 ± 0.04; *t*_90_ = 5.86, *p* < 0.00001). This difference may be attributed to the additional delay and/or perceptual disruption imposed by the IQ itself, or as we suggested above, to a possible response-repetition bias elicited during attention-congruent IQ trials. Given the attenuating effect of the IQ on the strength of AEs, and its possible role in obscuring attention-related effects, we designed Experiment 3 to focus primarily on No-IQ trials by including 75% No-IQ trials and only 25% IQ trials. The reason to include any IQ trials is that they provide an incentive for participants to follow the focusing instructions.

**FIGURE 5:**
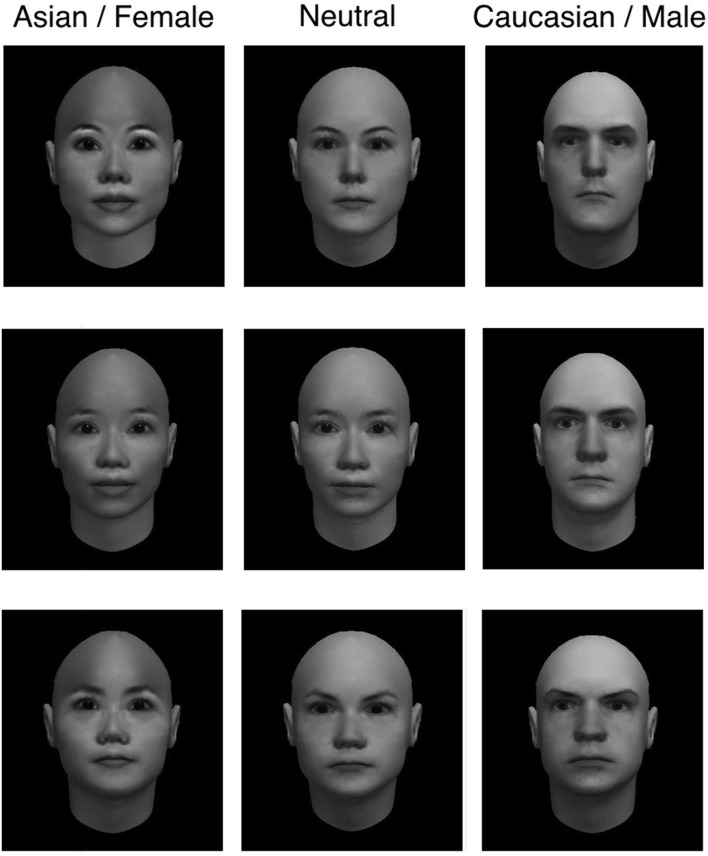
**Example stimuli from Experiment 3.** Neutral faces (shown in the second column) were constructed by generating perceptually neutral morphs between pairs of adapting faces (Asian female in the first column and Caucasian male in the third column).

Finally, we found in Experiment 2 just as in Experiment 1 that overall gender AEs (0.67 ± 0.05) were significantly stronger than race AEs (0.37 ± 0.04; *t*_90_ = 5.5, *p* < 0.00001). This difference cannot be attributed to differences in the unambiguity of the adaptors with respect to race and gender, which if anything should have led to slightly stronger race AEs (see **Figures 2A,B**). Instead, this bias may be due to the method by which the neutral faces were constructed. Due to the interdependence of facial dimensions in the face construction software, neutral faces had nonzero contributions of other races besides Asian and Caucasian. Therefore these neutral stimuli might not have served as ideal neutral test points to measure race AEs. We address this in Experiment 3 by constructing a new set of neutral faces that involved creating perceptually neutral morphs between pairs of adapting faces (see Methods/Stimuli; **Figure 5**).

### Experiment 3 Results

We present results for No-IQ trials, which constituted 75% of all trials. As we anticipated, the new method of generating neutral faces eliminated the discrepancy between race and gender AEs we had observed in Experiments 1 and 2. Specifically, we found that the overall magnitude of race AEs (0.81 ± 0.09) did not differ significantly from the overall magnitude of gender AEs (0.74 ± 0.10; *t*_53_ = 0.66, *p* > 0.5).

**FIGURE 6:**
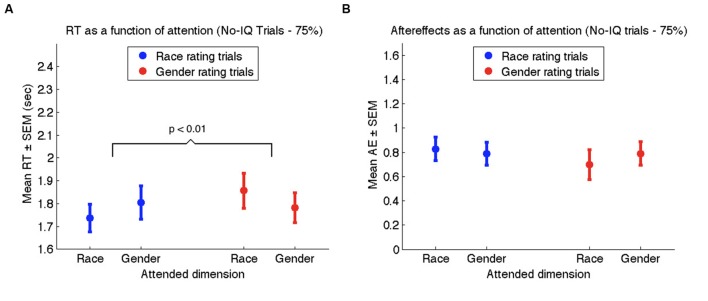
**Results of Experiment 3 for no-IQ trials only.** **(A)** Mean reaction times to the neutral face during race rating trials (blue) and gender rating trials (red) as a function of the attended dimension. **(B)** Mean aftereffect-consistent difference (AE) in ratings of race (blue) and gender (red) as a function of attended dimension during adaptation. Error bars denote standard error of the mean across 54 participants.

As in Experiments 1 and 2, reaction times in Experiment 3 were significantly influenced by the attention manipulation, wherein attention-congruent trials elicited shorter RTs (1.76 s ± 0.06) than attention-incongruent trials (1.83 s ± 0.07; *t*_53_ = 2.23, *p* < 0.01; see **Figure 6A**). Nevertheless, the strength of AEs once again was not influenced by attention (**Figure 6B**). Collapsing across race and gender rating trials, AEs on attention-congruent trials (0.81 ± 0.08) were similar to AEs on attention-incongruent trials (0.74 ± 0.09; *t*_53_ = 1.05, *p* > 0.2). Together with the results of Experiments 1 and 2, our data confirm that attending to the race or gender of a face does not influence the extent of adaptation to race or gender.

## Discussion

Our results show that race and gender AEs can be elicited in a single-shot adaptation paradigm, where both dimensions are adapted to simultaneously. Based on the results of Experiments 1 and 2, it seemed that gender AEs somehow dominated race AEs; however, a careful reconstruction of neutral stimuli in Experiment 3 based on directly morphing pairs of adapting faces eliminated this discrepancy and showed that race and gender AEs are comparable in magnitude.

Our data also show that soliciting a rating of the adapting face via an IQ can attenuate AEs due to a possible response-repetition bias that can occur only for attention-congruent trials. For example, a participant who responds “male” to an adapting face may inadvertently respond “male” again when asked about the gender of the neutral face, reducing the apparent magnitude of the AE. This repetition bias would not occur on attention-incongruent trials, where the participant is asked a question about race at test.

Across the three experiments, we showed when participants are asked to focus on the race or gender of an adapting face, their reaction times to rate a neutral face are influenced by the congruence of the two questions. This was the case in the presence or absence of an IQ, although it was stronger when the IQ reaffirmed the attended dimension. Despite this reaction time effect of attention, we found no evidence in any of our conditions that attending to race or gender increased the magnitude of AEs along that dimension. Across experiments, AEs during attention-incongruent trials were just as strong as (if not stronger than) AEs during attention-congruent trials.

It is possible that our attention manipulation did not provide enough incentive to participants to strongly focus on the race or gender of the faces. The validation measure for our attention manipulation was based on comparing reaction times to rate neutral faces during attention-congruent vs. attention-incongruent trials, with a greater reaction time during attention-incongruent trials indicating that the attention manipulation was successful. Even when the attention manipulation was successful in influencing reaction times, it is possible that the mechanism involved something other than encoding differences during adaptation. For example, participants may have been preparing to answer a question about race (or gender) rather than consciously attending to that attribute of the adapting face. While our data cannot rule out such an interpretation, the robustness of the attentional effect on response times across both IQ and NIQ trials suggests that participants were actually attending to the dimension as instructed. We note that the response time effect in Experiment 2 (where the IQ appeared on 50% of trials, and the mean reaction time difference was 165 ms ± 47 ms) trended to be stronger in Experiment 3 (where the IQ appeared on only 25% of trials, and the mean reaction time difference was 71 ms ± 32 ms, two-sample *t*_143_ = 1.77, *p* = 0.08). It is possible then that the presence of the IQ served as both an incentive to follow the attention instructions and a dampener of the adaptation effect. Therefore a future study may devise a stronger attention manipulation that can provide reliable incentives for participants to attend to relevant facial dimensions, without biasing or dampening the adaptation process itself.

Another possible reason we found no enhancement in attention-congruent AEs is that race and gender perception both involve similar and overlapping holistic processes (Tanaka et al., 2004; Zhao and Hayward, 2010). Because of the overlap in features that cue race and features that cue gender, perhaps observers cannot selectively attend to race cues while ignoring gender cues, or vice versa. For example, Ito and Urland (2003) conducted an event-related potential (ERP) study where participants were instructed to attend either to the race or gender of a sequence of faces. Although race-related activity was found to be greater and occur earlier than gender-related activity, both race and gender information were processed during the task, regardless of the attention condition. Furthermore, there is a general overlap between the facial areas used to make gender and race distinctions. Dupuis-Roy et al. (2009) showed that the eyebrow area contained the most gender information, while Blais et al. (2008) found that Western Caucasian observers fixated on the eye and mouth regions when determining the race of a face, though they also found a significant cultural difference, with East Asian observers focusing more centrally when determining race. Indeed, even among participants of European origin, there were differences in the eye movements elicited by same- and other-race face (Brielmann et al., 2014). In our studies, participants were free to inspect the faces freely during the 5-s adaptation phases, so a future eye tracking study may illuminate whether attending to race vs. gender results in different scan paths, and in turn, different adaptation mechanisms for some observers.

There is evidence in other visual domains that attending differentially to the same stimulus can lead to different AEs. For example, Lankheet and Verstraten (1995) showed that the direction of motion AEs can be modulated by attention. Participants were asked to view a moving random dot pattern that contained both a leftward and rightward motion signal, each of which could be selectively attended to. The authors found that if one or the other direction was attended to during adaptation, a corresponding motion AE was observed. In a later study, Spivey and Spirn (2000) investigated the tilt AE in a similar fashion. Normally, adapting to tilted bars in a particular direction of tilt causes a subsequent vertical bar to appear tilted in the opposite direction. In Spivey and Spirn’s (2000) study, the adapting stimuli contained overlapping tilted bars rotated clockwise and counterclockwise. The authors found when participants were instructed to attend to the clockwise tilted bars, the direction of the tilt AE was in the counterclockwise direction (and vice versa). Both of these studies demonstrate that in lower level visual processes, attending to a specific dimension of a stimulus can drive differential AEs with respect to that dimension. It remains to be shown why such attention-dependent AEs are not observed in the perception of race and gender.

## Author Contributions

ND was primarily responsible for the conception of the project, development of the adaptation paradigm, analysis of data, and writing the methods, results, and discussion of the manuscript. CV was critically involved in the construction and validation of the adapting and neutral face stimuli and coding experimental scripts. NH wrote the first draft of the introduction and contributed along with CV to interpreting results and validating the face stimuli. JC helped in stimulus design by testing the efficacy of different formats of face images in eliciting aftereffects using web surveys. All authors contributed to the editing of the final draft.

## Conflict of Interest Statement

The authors declare that the research was conducted in the absence of any commercial or financial relationships that could be construed as a potential conflict of interest.

## Footnotes

1 http://www.facegen.com/
